# Sonographic Diagnosis of Flexor Tendon Incarceration by a Malunited Fracture Fragment: A Case Report

**DOI:** 10.3390/diagnostics16091260

**Published:** 2026-04-23

**Authors:** Yuan-Chen Chang, Yu-Te Lin, Yu-Hsuan Cheng

**Affiliations:** 1 Department of Physical Medicine and Rehabilitation, Wan Fang Hospital, Taipei Medical University, Taipei 116, Taiwan; 112131@w.tmu.edu.tw; 2 Department of Plastic and Reconstructive Surgery, Chang Gung Memorial Hospital, Taoyuan 333, Taiwan; linutcgmh@gmail.com; 3 International Master Science Program in Reconstructive Microsurgery, Chang Gung University, Taoyuan 333, Taiwan; 4 Department of Physical Medicine and Rehabilitation, School of Medicine, College of Medicine, Taipei Medical University, Taipei 116, Taiwan

**Keywords:** case report, finger stiffness, ultrasound, post-fracture malunion, tendon incarceration

## Abstract

**Background and Clinical Significance**: Post-traumatic finger stiffness is frequently attributed to soft tissue adhesions; however, mechanical obstruction from occult osseous structures remains a rare but critical differential diagnosis in adults. **Case Presentation**: This report describes a 56-year-old female presenting with severe, refractory stiffness of the little finger eight months after a proximal phalanx fracture. Despite extensive conservative therapy, active and passive flexion at the proximal and distal interphalangeal joints remained locked in extension. While conventional radiographs demonstrated bony union, musculoskeletal ultrasonography (MSUS) revealed an occult protruding malunited fragment incarcerating the flexor tendons. Dynamic MSUS provided real-time evidence of mechanical impingement by demonstrating proximal muscle contraction without distal tendon excursion. Intraoperatively, initial soft tissue tenolysis failed to restore motion; further exploration guided by MSUS evidence successfully identified a sharp bone spike. Subsequent ostectomy resulted in immediate restoration of functional range of motion. This case underscores the limitations of static imaging in evaluating the dynamic gliding mechanism and highlights the valuable role of MSUS in identifying mechanical functional obstructions. **Conclusions**: Early sonographic evaluation should be considered for refractory post-traumatic stiffness to prevent prolonged, ineffective conservative care and to guide definitive surgical management.

## 1. Introduction

Post-traumatic joint stiffness is a common yet challenging complication in musculoskeletal rehabilitation. Owing to the constricted fibro-osseous canals and complex tendon-gliding mechanisms, the fingers are particularly susceptible to severe stiffness following trauma. Proximal phalanx fractures, in particular, frequently result in a stiff digit. Although the etiology is multifactorial, including soft tissue, articular, and iatrogenic factors, clinical restriction of motion is most commonly attributed to peritendinous adhesions or joint contractures [1]. Because soft tissue adhesion is usually the presumed cause, mechanical obstruction from a bony block is often overlooked and remains exceptionally rare in adults.

Nevertheless, an occult bony prominence must be carefully ruled out in refractory cases, as missed diagnoses can lead to attritional tendon rupture without prompt surgical intervention. Although plain radiography is routinely used for initial evaluation, it cannot adequately visualize the dynamic bone–tendon interface. Ultrasonography overcomes this limitation by assessing both static tendon architecture and dynamic gliding function. Herein, we present a rare adult case of flexor tendon incarceration caused by an occult malunited fracture fragment, highlighting the valuable role of dynamic musculoskeletal ultrasonography (MSUS) in identifying mechanical functional obstruction and guiding definitive surgical management.

## 2. Case Report

**Figure 1:**
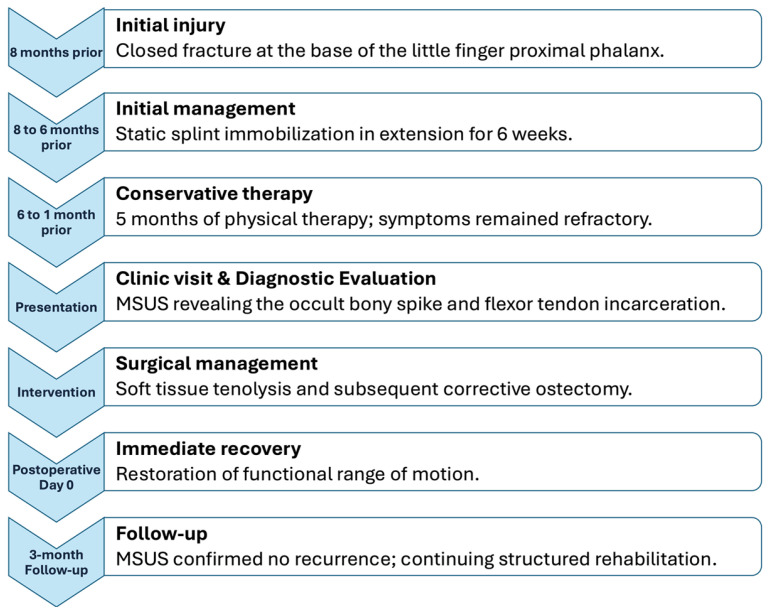
Clinical timeline of the diagnostic and therapeutic course.

**Figure 2:**
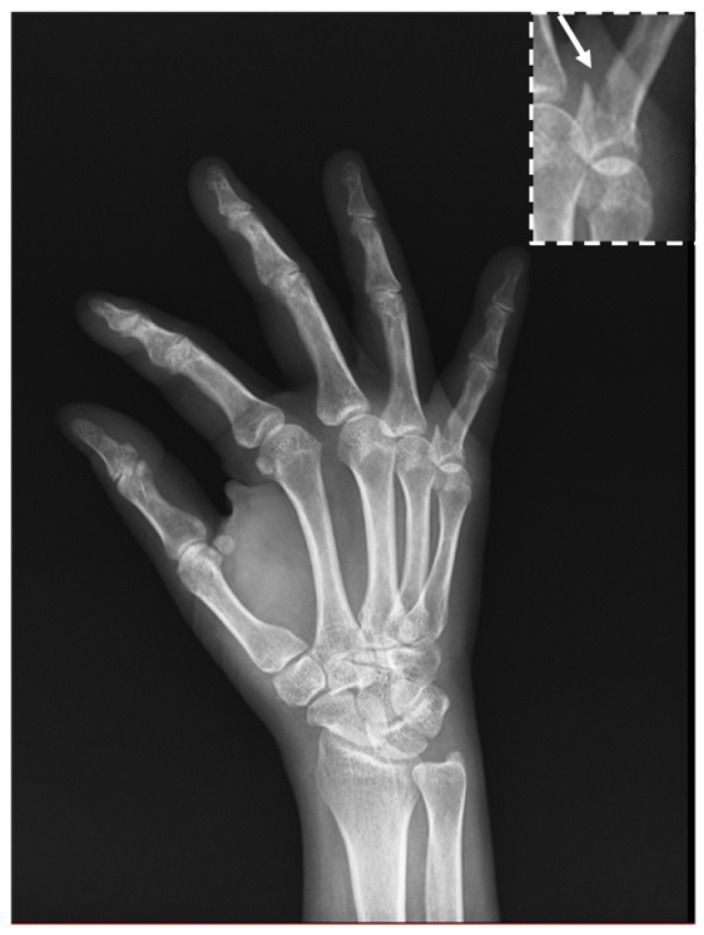
Initial lateral view demonstrates a fracture at the base of the proximal phalanx of the little finger (arrow). The magnified inset provides a clearer visualization of the fracture site.

A 56-year-old right-handed woman presented to our rehabilitation clinic with marked stiffness and functional impairment of her right little finger. The patient’s medical history was unremarkable for systemic conditions, such as diabetes mellitus or autoimmune diseases, and she had no smoking history or pre-existing trauma to the right hand prior to the injury described in this report. The clinical course and management are summarized in Figure 1. Eight months prior to presentation, she had sustained a closed fracture at the base of the proximal phalanx of the right little finger (Figure 2). The injury was initially managed conservatively with static splint immobilization in extension for six weeks. Following splint removal, she developed persistent limitation of finger motion. The stiffness was initially interpreted as a routine sequela of prolonged immobilization. Despite undergoing five months of conservative physical therapy, including therapeutic ultrasound, hot packing, and acupuncture, her symptoms remained refractory.

Physical examination revealed painless but severe restriction of both active and passive flexion at the proximal interphalangeal (PIP) and distal interphalangeal (DIP) joints. The active range of motion (ROM) was severely limited to nearly 0° of flexion at both the PIP and DIP joints, rendering the finger locked in extension. Passive ROM was similarly restricted, exhibiting a firm, abrupt end-feel. There was no focal tenderness over the volar aspect of the digit, and sensorimotor examination was unremarkable.

**Figure 3:**
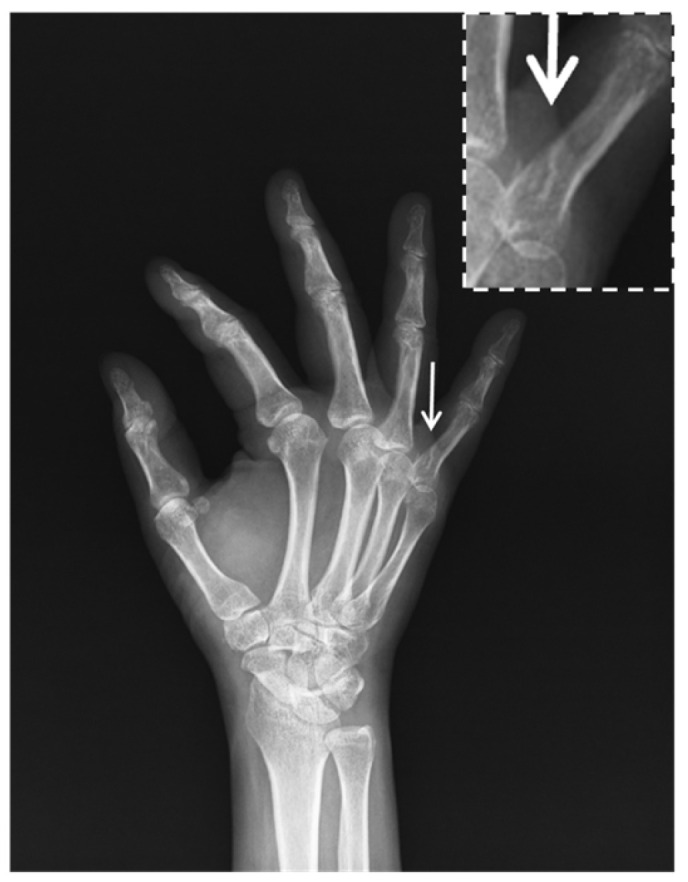
Follow-up lateral view obtained three months later, showing fracture union. The magnified inset highlights the fracture union (arrow).

Follow-up radiographs confirmed solid bony union of the proximal phalanx with a relatively smooth contour (Figure 3), lacking any obvious mechanical bony obstruction that could account for the persistent stiffness.

Given the persistent finger stiffness, several differential diagnoses including tendon rupture, post-traumatic tendon adhesion, and joint contracture were considered. Tendon rupture was less likely because the patient retained active finger flexion tension in small range of motion, and no palpable gaps were noted during examination. Soft-tissue adhesions were the primary initial suspicion following the period of immobilization; however, the total lack of clinical progress suggested a more definitive mechanical obstruction. Joint contracture was also considered, but the firm, abrupt end-feel during passive movement pointed toward a mechanical block rather than simple capsular tightness. Since the follow-up radiographs (Figure 3) showed a relatively smooth bony union, an occult bony spike or dynamic tendon incarceration remained high on the list of suspicions.

**Figure 4:**
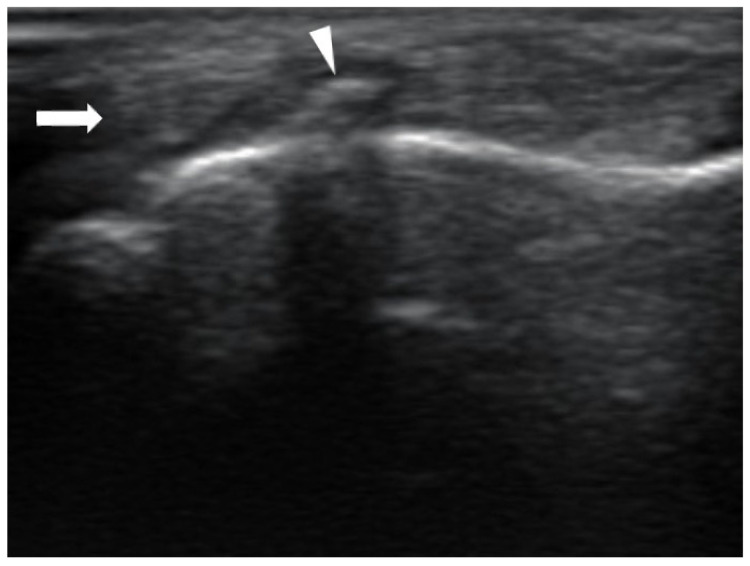
Ultrasound image of the little finger in longitudinal view. The arrowhead points to the bony spike arising from the fracture site of the proximal phalanx. The arrow indicates the flexor tendon being incarcerated and mechanically impinged by the bony protrusion.

To further evaluate the soft tissue and dynamic gliding mechanism, high-resolution musculoskeletal ultrasonography was performed using a tablet-based ultrasound system (minisono L3-12, ALPINION Medical Systems, Seoul, Republic of Korea). Sonographic evaluation revealed a sharp, vertically oriented bony spike arising from the volar aspect of the healed fracture site, protruding directly into the flexor tendon sheath (Figure 4). The fibrillar architecture of the flexor tendon was preserved without any anechoic gaps, ruling out a tendon tear. During attempted active flexion, dynamic sonographic assessment demonstrated proximal muscle contraction without any distal tendon excursion at the fracture site. During passive flexion, the tendon was observed to directly impinge against the bony spike.

**Figure 5:**
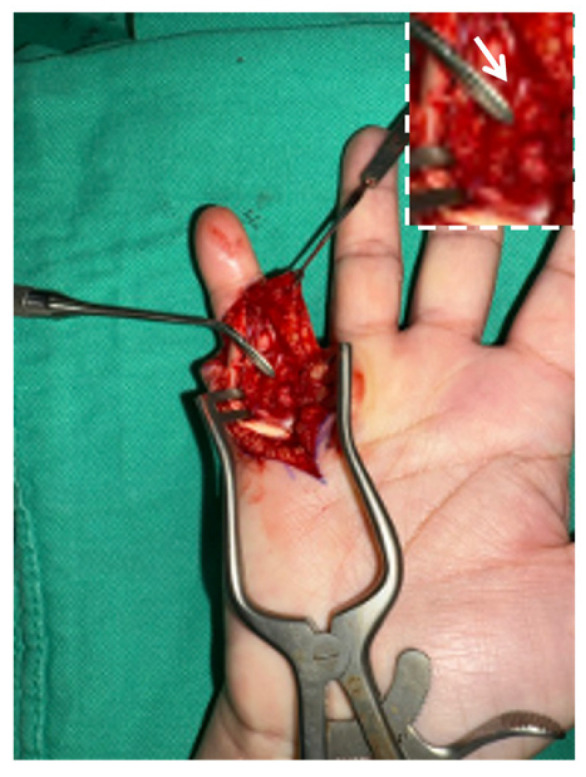
Intraoperative image of right little finger demonstrating a protruding bone prominence (forceps tip) incarcerating the flexor digitorum profundus and disrupting the flexor digitorum superficialis radial slip. The magnified inset provides a detailed view of the offending bone spike (arrow).

Based on the sonographic evidence of severe peritendinous adhesions and/or mechanical incarceration, the patient was referred for surgical intervention. Tenolysis was performed under local anesthesia. Intraoperatively, the surgical team initially performed a thorough soft tissue tenolysis; however, finger motion remained restricted. Guided by the sonographic evidence, further exploration identified a sharp, protruding bone prominence incarcerating the flexor digitorum profundus tendon and disrupting the radial slip of the flexor digitorum superficialis (Figure 5). The protruding bone prominence was subsequently resected via ostectomy, and the damaged tendon tissue was debrided. Postoperatively, the patient experienced immediate improvement in digit mobility, with PIP and DIP joint flexion recovering to 90° and 50°, respectively, restoring functional grasp.

Immediately following the procedure, a light dressing was applied to facilitate early mobilization, and encourage maximal tendon excursion and prevent recurrent adhesions. A structured rehabilitation program was initiated after surgery. The protocol focused on edema control, active-assisted ROM, and tendon-gliding exercises. Recovery was monitored through monthly clinical follow-ups, during which functional ROM and pain intensity were recorded.

The patient was followed for three months postoperatively. At the most recent clinical visit, she expressed high satisfaction with the surgical outcome, noting that the restored ability to grasp objects had significantly improved her quality of life. The functional range of motion achieved immediately after surgery (PIP 90° and DIP 50°) was successfully maintained. Although mild discomfort persisted during end-range finger flexion, with a pain intensity of 3/10 on the Visual Analog Scale, follow-up MSUS demonstrated restored flexor tendon gliding with no evidence of recurrent bony incarceration. To further optimize functional outcomes and address the residual end-range pain, she is currently continuing a structured rehabilitation program including thermal therapy and passive joint mobilization.

Written informed consent for publication was obtained from the patient following approval by the local institutional review board (Taipei Medical University Joint Institutional Review Board, N202602015).

## 3. Discussion

### 3.1. Epidemiology and Pathophysiology in Post-Fracture Tendon Incarceration

Post-fracture stiffness of the digits is a common clinical challenge, typically resulting from joint contractures or peritendinous adhesions due to immobilization [1,2]. While tendon entrapment following phalangeal fractures is well-documented in the pediatric population [3,4,5,6,7,8,9,10], where the unmineralized physis serves as a biomechanical weak point [7] for acute “buttonhole” mechanisms during fracture displacement [10], this specific mechanism is eliminated upon skeletal maturity [11].

The pathophysiology in this adult case is characterized by a volar bony prominence post-fracture, distinct from cases where the flexor tendon is entrapped due to herniation into the fracture gap [12]. Due to the anatomical proximity of the flexor tendon sheath to the volar cortex of the proximal phalanx [13,14], particularly the flexor digitorum profundus [10], even minor osseous alterations can severely incarcerate the gliding mechanism. When conservative measures fail to restore mobility, clinicians must maintain a high index of suspicion for a mechanical osseous block to avoid prolonged, ineffective therapy and the potential risk of attritional tendon rupture [15,16]. Although several reports of post-fracture tendon incarceration in adults exist in the literature, the routine utilization of MSUS for definitive diagnosis remains limited [12,17].

### 3.2. Diagnostic Challenges and Clinical Application of High-Resolution MSUS

This case highlights the diagnostic limitations of conventional static imaging modalities. Although standard radiography is recommended as the first-line investigation to exclude osseous blocks [18], identifying entrapment at the proximal phalanx base is uniquely challenging due to the anatomical superimposition of the metacarpal head. Unlike prominent shaft-level spikes, these minute and avulsion-related bony prominences are easily misidentified as benign osteophytes or simple cortical thickening on plain films, particularly when not projected in a true tangential plane. Furthermore, while advanced static imaging such as computed tomography (CT) or magnetic resonance imaging (MRI) can delineate detailed anatomical structures, they may be insufficient for functional entrapment that is only visible during active tendon excursion. To precisely localize the mechanical entrapment, dynamic sonographic monitoring was utilized after ensuring tendon continuity. We evaluated the bone–tendon interface through both passive distal gliding and active proximal muscle contraction. These real-time observations verified that the site of restricted tendon excursion corresponded exactly to the location of the volar bony spike.

High-resolution MSUS effectively overcame these limitations in this case by providing three key insights that directly influenced our clinical decision-making: (1) excluding a complete flexor tendon rupture by confirming the continuity of the fibrillar architecture; (2) visualizing the precise location of the occult volar malunited fragment; and (3) confirming the precise site of mechanical impingement by identifying the point where tendon gliding was disrupted during both active and passive maneuvers. This dynamic evidence of a mechanical block provided diagnostic clarity that could not be achieved through clinical examination or static imaging alone.

### 3.3. Differential Diagnosis Based on MSUS

Clinically distinguishing post-fracture soft tissue adhesions from true mechanical incarceration is challenging, as both conditions present with a reduction in active range of motion. On MSUS, simple adhesions typically demonstrate loss of defined linear tendon margins, peritendinous soft tissue encasement, synovial sheath thickening, and restricted yet preserved tendon motion during dynamic evaluation [19,20]. In contrast, true mechanical incarceration exhibits a distinct signature characterized by a complete or near-total absence of distal tendon excursion despite visible proximal muscle contraction. By visualizing the precise mechanical impingement between the tendon and the malunited bony fragment, dynamic MSUS provides an additional diagnostic clarity that complements clinical examination and static imaging.

### 3.4. Clinical Impact

Identifying a true osseous block has significant surgical implications. Mechanical incarceration due to bone is often refractory to both conservative rehabilitation and isolated soft-tissue tenolysis. This was evidenced intraoperatively in our patient, where tenolysis alone failed to restore finger flexion. Targeted exploration, guided by preoperative sonographic evidence, led to the identification of the offending bony prominence and subsequent successful ostectomy. Therefore, preoperative sonographic evaluation directly informs surgical strategy, shifting the approach from a simple soft-tissue release to a combined procedure involving osseous resection.

### 3.5. Study Limitations

There are several limitations to this report that warrant consideration. First, the single-case design inherently limits the generalizability of the findings to all types of proximal phalanx fractures. Second, advanced preoperative cross-sectional imaging, such as MRI or CT, was not performed; however, dynamic MSUS provided sufficient evidence for surgical planning. Third, the absence of recorded dynamic video clips during the initial evaluation is a limitation, although real-time findings were definitively documented. Additionally, MSUS is inherently operator-dependent, requiring specific technical expertise to identify occult mechanical blocks. Finally, while the patient showed significant improvement, the three-month follow-up period is relatively short and may not capture long-term functional stability. Despite these constraints, the strong correlation between sonographic findings and intraoperative evidence supports the clinical utility of MSUS in managing refractory post-traumatic finger stiffness.

## 4. Conclusions

In conclusion, this case illustrates that post-fracture finger stiffness in adults, although commonly attributed to soft tissue adhesions, may also result from rare mechanical obstructions such as flexor tendon incarceration. When severe restriction of both active and passive motion is refractory to standard rehabilitation, clinicians should consider the possibility of occult bony malunion. Dynamic MSUS is a valuable, real-time diagnostic modality for identifying functional tendon incarceration caused by occult post-fracture malunited fragments. Early sonographic evaluation should be considered in similar clinical scenarios to prevent prolonged, ineffective conservative treatment and to directly guide definitive surgical management involving corrective ostectomy.

## Data Availability

All data generated or analyzed during this study are included in this published article.

## Abbreviations

The following abbreviations are used in this manuscript:

CT	Computed tomography
DIP	Distal interphalangeal joint
MRI	Magnetic resonance imaging
MSUS	Musculoskeletal ultrasonography
PIP	Proximal interphalangeal joint
ROM	Range of motion
